# Supplementary material for: Temporal and spatial modulation of the tumor and systemic immune response in the murine Gl261 glioma model
Source: PLoS One. 2020 Apr 2;15(4):e0226444. doi: 10.1371/journal.pone.0226444 (PMC7117758; doi:10.1371/journal.pone.0226444)

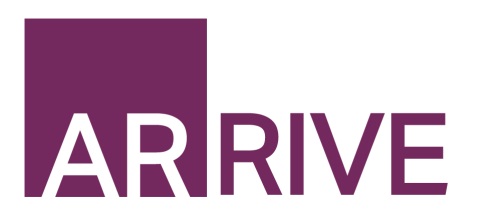


The ARRIVE Guidelines Checklist

Animal Research: Reporting In Vivo Experiments

Carol Kilkenny^1^, William J Browne^2^, Innes C Cuthill^3^, Michael Emerson^4^ and Douglas G Altman^5^

*^1^The National Centre for the Replacement, Refinement and Reduction of Animals in Research, London, UK, ^2^School of Veterinary Science, University of Bristol, Bristol, UK, ^3^School of Biological Sciences, University of Bristol, Bristol, UK, ^4^National Heart and Lung Institute, Imperial College London, UK, ^5^Centre for Statistics in Medicine, University of Oxford, Oxford, UK.*

|  | | ITEM | RECOMMENDATION | Section/ Paragraph |
| --- | --- | --- | --- | --- |
| 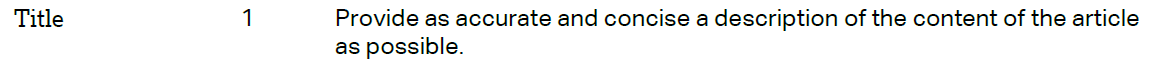 | | | Title |  |
| 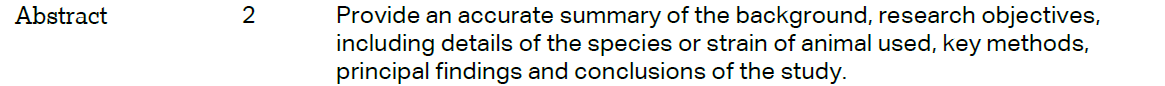 | | | Abstract |  |
| INTRODUCTION | | |  |  |
| 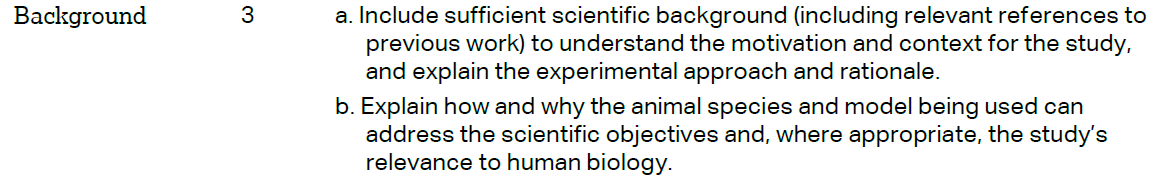 | | | Paragraph 1-2  Paragraph 3 |  |
| 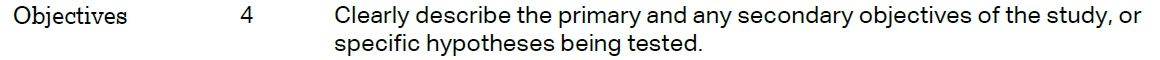 | | | Paragraph 4 |  |
| METHODS | | |  |  |
| 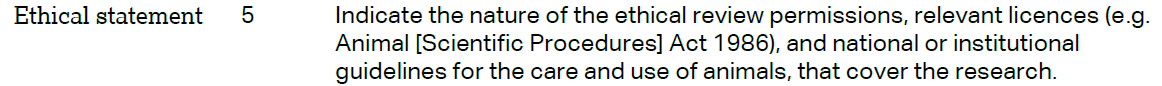 | | | Mice subsection, paragraph 1 |  |
| 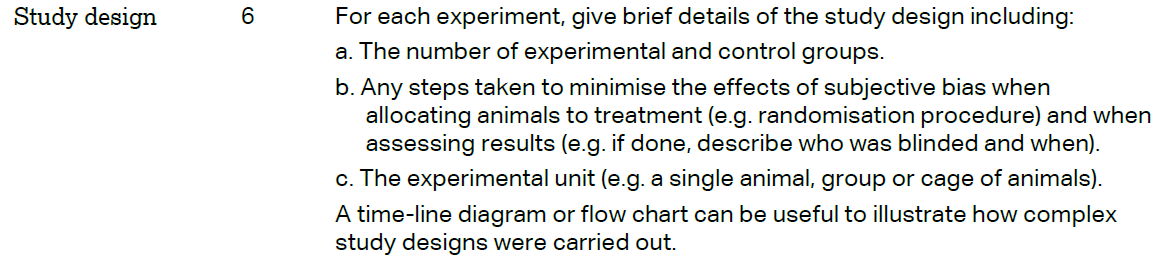 | | | Mice subsection, paragraph 2-3 |  |
| 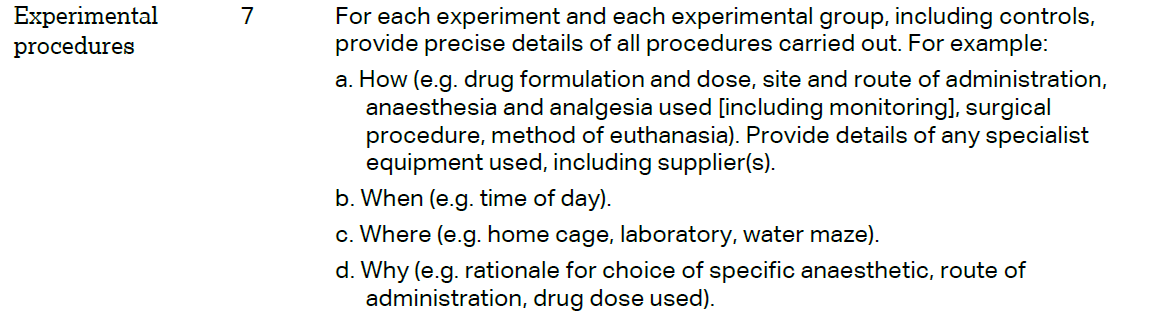 | | | Mice subsection, paragraph 3 |  |
| 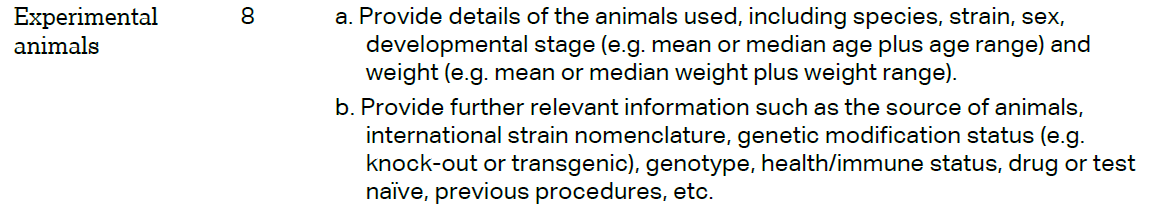 | | | Mice subsection, paragraph 2 |  |

The ARRIVE guidelines. Originally published in *PLoS Biology*, June 2010^1^

| 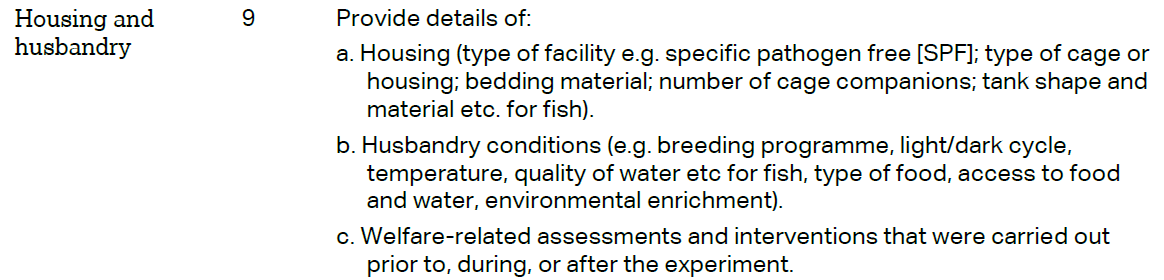 | Mice subsection, paragraph 2 | |
| --- | --- | --- |
| 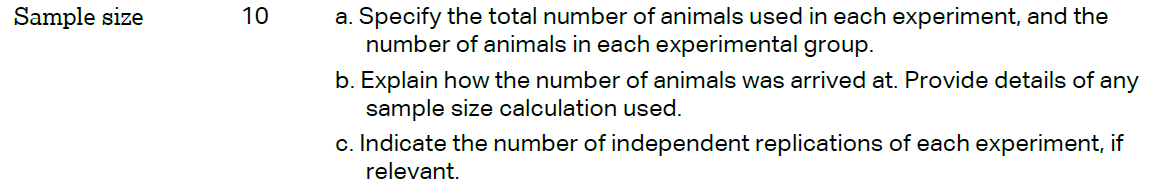 | Mice subsection, paragraph 3 | |
| 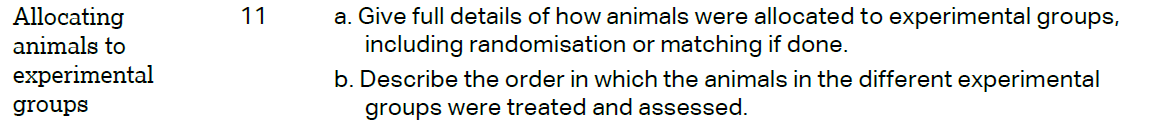 | Mice subsection, paragraph 3 | |
| 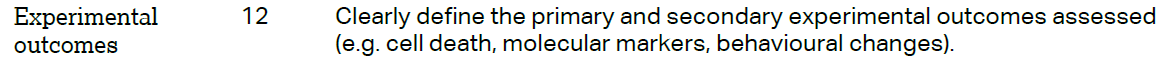 | Intro paragraph 4. | |
| 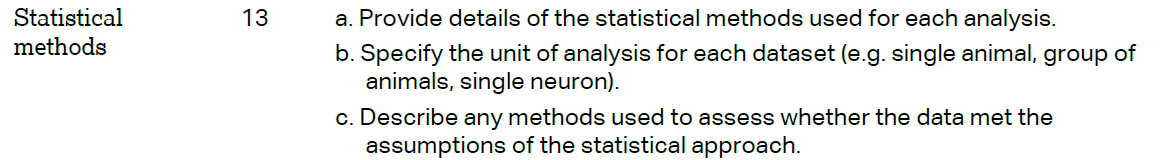 | Statistical analyses subsection | |
| RESULTS |  | |
| 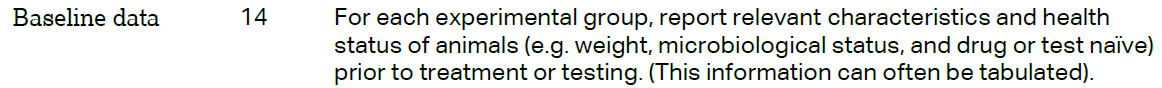 | Methods mice subsection paragraph 3. | |
| 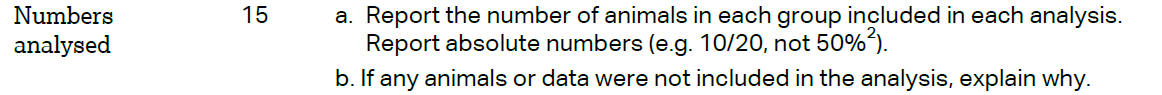 | Figure captions. | |
| 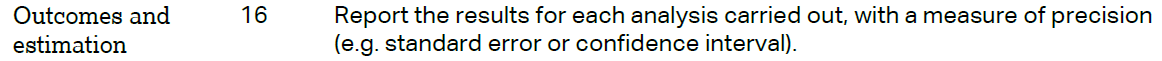 | Results paragraphs 1-11. | |
| 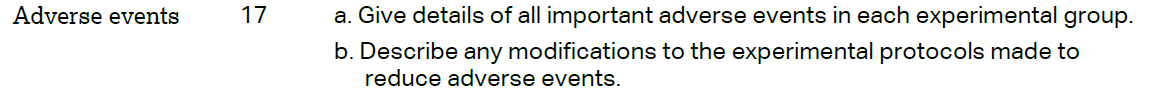 | Methods mice subsection, paragraph 3 | |
| DISCUSSION |  | |
| 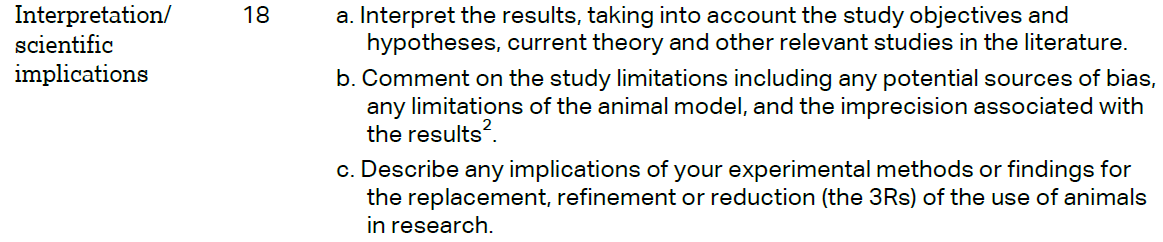 | Discussion paragraph 2-5. | |
| 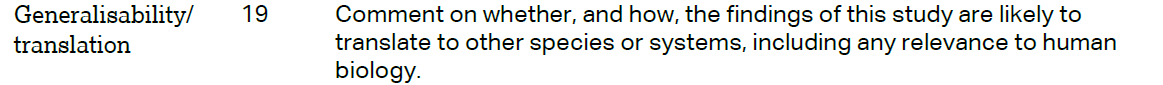 | Conclusion paragraph 1. | |
| 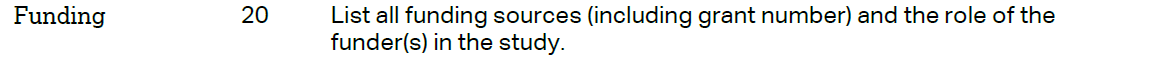 | | Funding subsection |


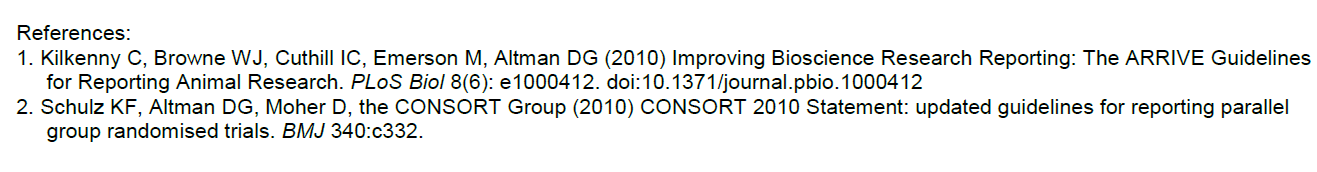

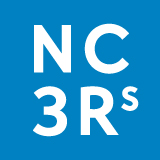

Supplement: S1 Checklist — (DOCX) [file pone.0226444.s001.docx]
