# Supplementary figures and images for: Temporal and spatial modulation of the tumor and systemic immune response in the murine Gl261 glioma model
Source: PLoS One. 2020 Apr 2;15(4):e0226444. doi: 10.1371/journal.pone.0226444 (PMC7117758; doi:10.1371/journal.pone.0226444)

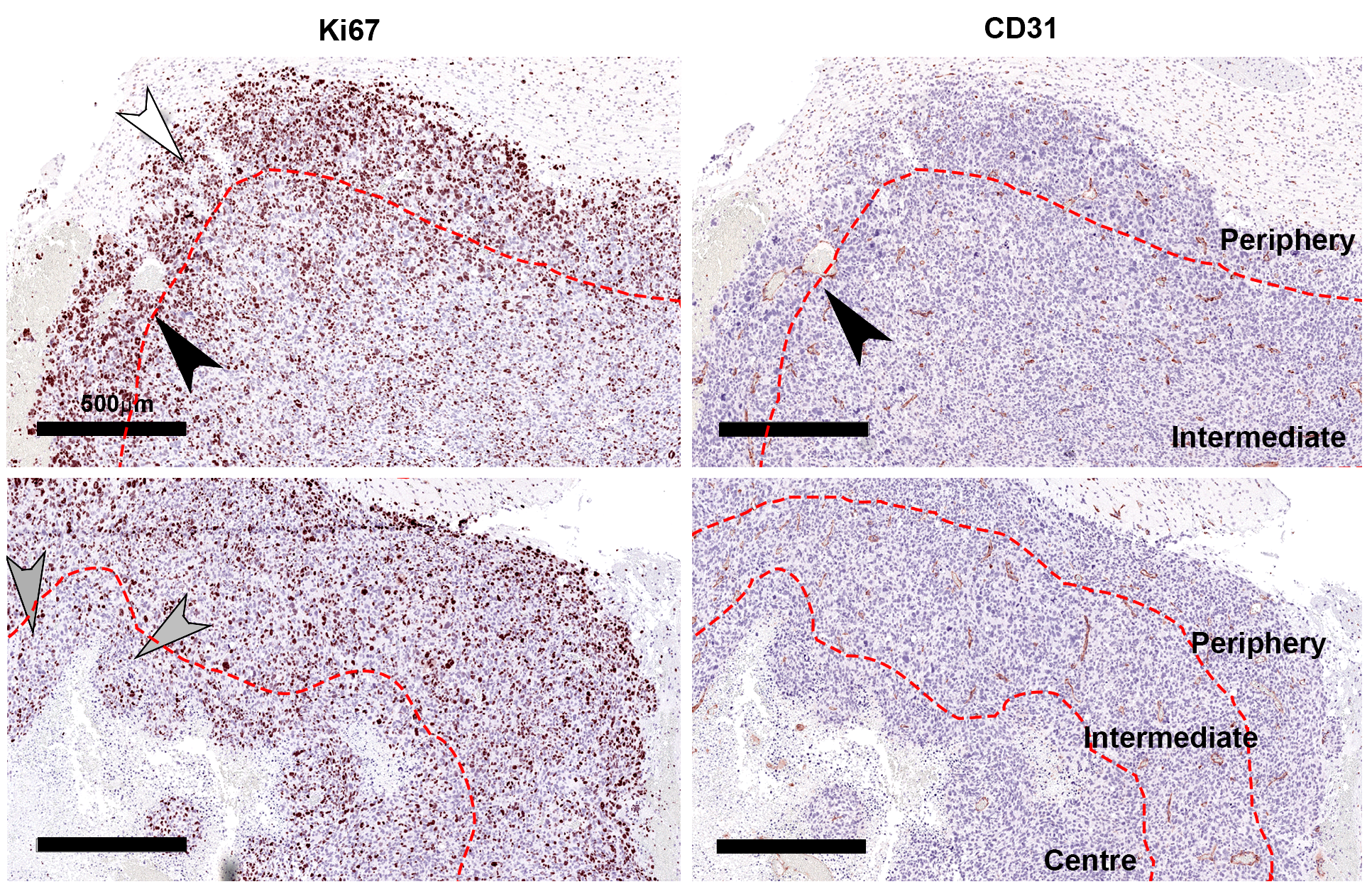

Supplement: S1 Fig — (TIF) [file pone.0226444.s002.tif]

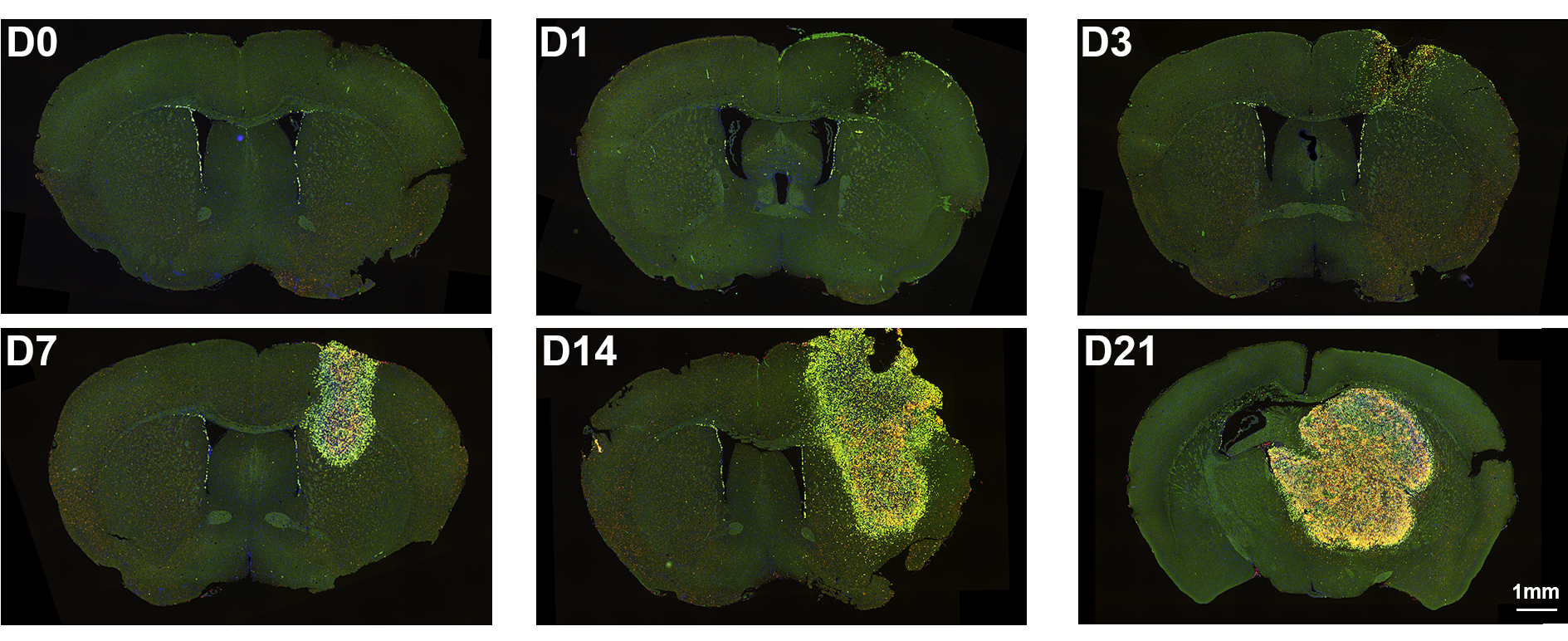

Supplement: S3 Fig — (TIF) [file pone.0226444.s004.tif]

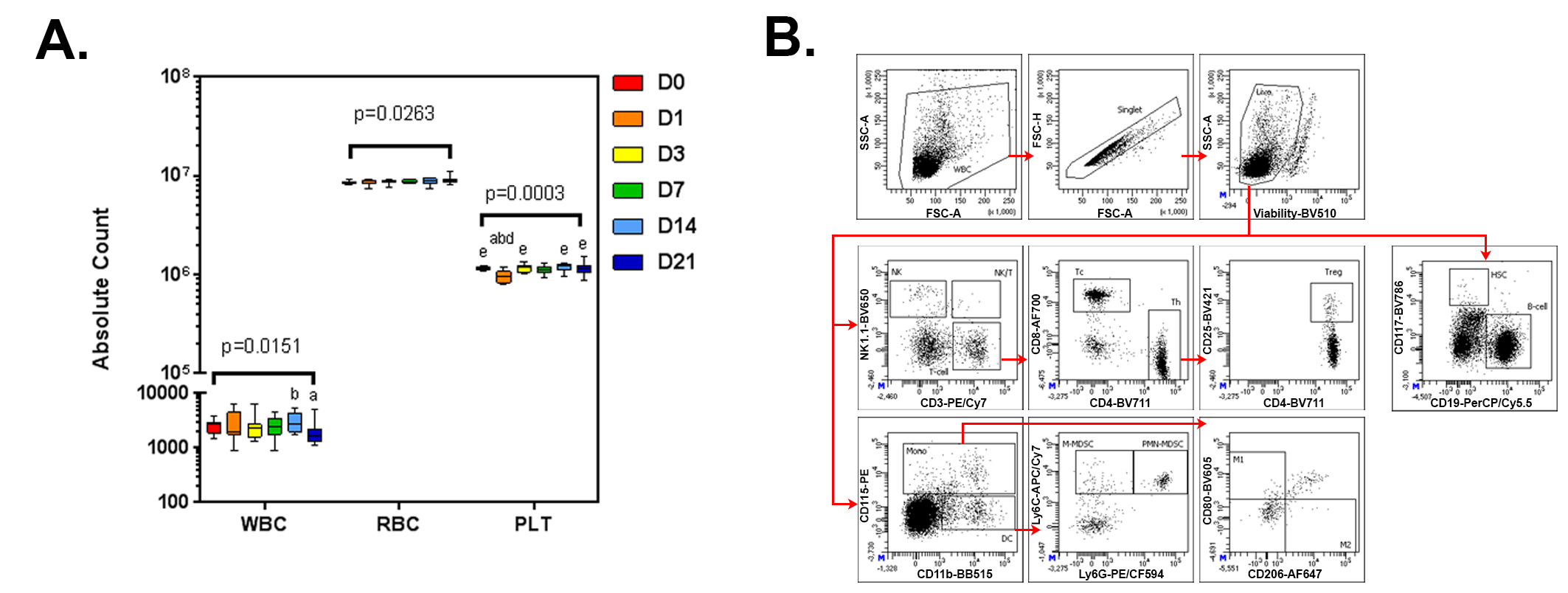

Supplement: S4 Fig — (TIF) [file pone.0226444.s005.tif]

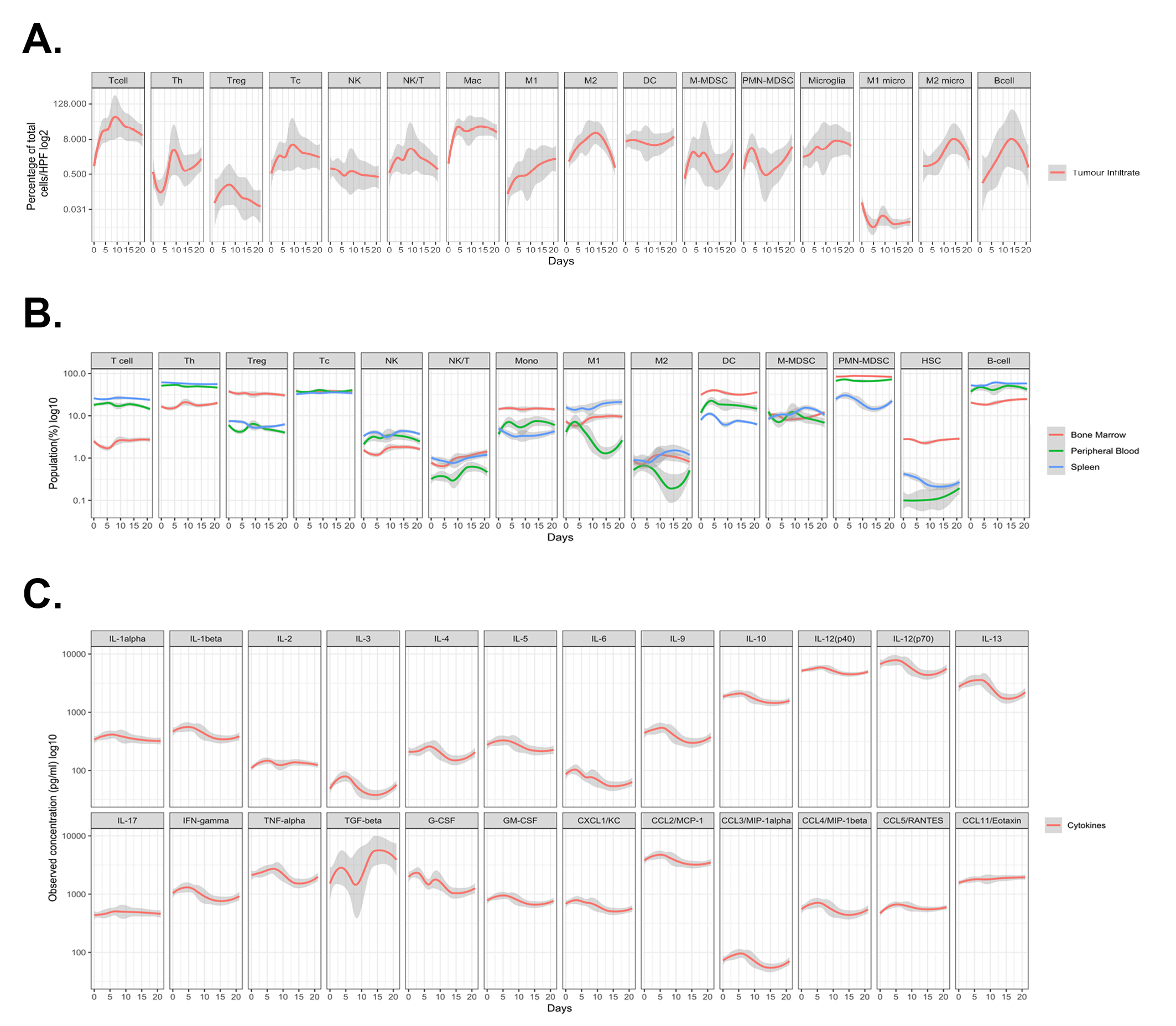

Supplement: S5 Fig — (TIF) [file pone.0226444.s006.tif]
